# Cortico-thalamic development and disease: From cells, to circuits, to schizophrenia

**DOI:** 10.3389/fnana.2023.1130797

**Published:** 2023-03-02

**Authors:** Marilyn M. Angulo Salavarria, Claudia Dell’Amico, Armando D’Agostino, Luciano Conti, Marco Onorati

**Affiliations:** ^1^Unit of Cell and Developmental Biology, Department of Biology, University of Pisa, Pisa, Italy; ^2^Department of Health Sciences, University of Milan, Milan, Italy; ^3^Department of Mental Health and Addictions, ASST Santi Paolo e Carlo, Milan, Italy; ^4^Department of Cellular, Computational, and Integrative Biology, University of Trento, Trento, Italy

**Keywords:** cerebral cortex, thalamus, schizophrenia, organoids, neurodevelopment

## Abstract

The human brain is the most complex structure generated during development. Unveiling the ontogenesis and the intrinsic organization of specific neural networks may represent a key to understanding the physio-pathological aspects of different brain areas. The cortico-thalamic and thalamo-cortical (CT-TC) circuits process and modulate essential tasks such as wakefulness, sleep and memory, and their alterations may result in neurodevelopmental and psychiatric disorders. These pathologies are reported to affect specific neural populations but may also broadly alter physiological connections and thus dysregulate brain network generation, communication, and function. More specifically, the CT-TC system is reported to be severely affected in disorders impacting superior brain functions, such as schizophrenia (SCZ), bipolar disorder, autism spectrum disorders or epilepsy. In this review, the focus will be on CT development, and the models exploited to uncover and comprehend its molecular and cellular mechanisms. In parallel to animal models, still fundamental to unveil human neural network establishment, advanced *in vitro* platforms, such as brain organoids derived from human pluripotent stem cells, will be discussed. Indeed, organoids and assembloids represent unique tools to study and accelerate fundamental research in CT development and its dysfunctions. We will then discuss recent cutting-edge contributions, including *in silico* approaches, concerning ontogenesis, specification, and function of the CT-TC circuitry that generates connectivity maps in physiological and pathological conditions.

## Cortico-thalamic and thalamo-cortical development according to the prosomeric model

The human brain is a sophisticated combination of circuit interaction with multiple degrees of complexity. The specification of distinct types and subtypes of neurons requires the execution of elaborated region-specific differentiation programs that progressively instruct neural progenitors toward maturity (Rakic, 2009; Clascá et al., 2012; Silbereis et al., 2016; Li et al., 2018, 2020). Specific intrinsic and extrinsic molecular mechanisms modulate networks ontogenesis in order to establish the appropriate communication between brain areas (O’Leary and Koester, 1993; López-Bendito and Molnár, 2003; Faingold and Blumenfeld, 2014; Cadwell et al., 2019). Most of our knowledge about the key mechanisms regulating brain region-specific ontogenesis is coming from studies on animal models, very precious and representative thanks to the evolutionary conservation of fundamental developmental pathways (Shi et al., 2021).

The CT-TC circuitry represents a pertinent example of an elaborated network that regulates brain sensory processing, learning and memory, sleep, plasticity, and consciousness (López-Bendito, 2018). The cerebral cortex and the thalamus operate as a close unit (Cadwell et al., 2019) and the establishment of their reciprocal connections initiates at early developmental stages – observed in the human embryo at 7.5/8 postconceptional weeks (pcw) and in the marmoset monkey at embryonic day (E) 55 (Alzu’Bi et al., 2019). In particular, these connections appear soon after thalamus and pre-thalamus formation, when distance is minimal, mutually influencing their development (Molnár and Blakemore, 1995; Grant et al., 2012; Alzu’Bi et al., 2019).

**FIGURE 1:**
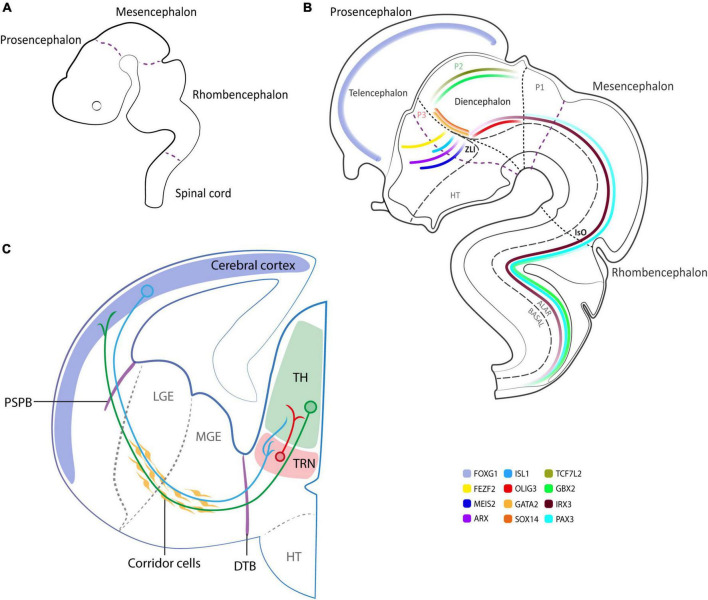
Schematics of human neurodevelopment. **(A)** Neuromere formation according to the prosomeric model at 3 brain vesicle stage (3–4 pcw). **(B)** Schematics at 5 brain vesicle stage, illustrating the expression pattern of relevant neurodevelopmental genes with special emphasis on the diencephalon (6 pcw). **(C)** Schematics of the cortico-thalamic (CT) and thalamo-cortical (TC) projections in the developing human brain (9–10 pcw). In blue and green representative CT and TC neurons, respectively, cross PSPB and DTB to reach their target area. In red TRN GABAergic neurons projecting to specific thalamic nuclei. P1, P2, P3, prosomere 1, 2, 3; ZLI, zona limitans intrathalamica; IsO, isthmic organizer; PSPB, pallial-subpallial boundary; LGE, lateral ganglionic eminence; MGE, medial ganglionic eminence; DTB, diencephalic-telencephalic boundary; TH, thalamus; TRN, thalamic reticular nucleus; HT, hypothalamus.

Both structures derive from a single brain vesicle, the prosencephalon. During neural development, different morphogens or master regulators [such as Bone Morphogenic Protein (BMP); Sonic Hedgehog (SHH); Wingless-related integration site (WNT); Fibroblast Growth Factors (FGF); Retinoic Acid (RA), etc.] contribute to correctly specify the rostro-caudal axis, and subsequently the dorso-ventral pattern (Rubenstein and Beachy, 1998; Ten Donkelaar et al., 2014). These macro-areas undergo further specifications and the most accredited theory that delineates their compartmentalization is the prosomeric model, postulated by Puelles and Rubenstein (1993) and Rubenstein et al. (1994), and further strengthened over the years. This model relies on the combinatorial action of genes that exhibit temporally and spatially restricted expression patterns (Figures 1A, B). This postulated segmental structural model, in contrast to the columnar model introduced by Herrick (1910), depicts the brain as an uninterrupted series of transverse subunits and emphasizes evolutionarily conserved topological and molecular relationships along the neural tube. These transverse subunits (or segments) are generally called neuromeres and subdivide the prosencephalon, the mesencephalon and the rhombencephalon (Figure 1A; Puelles and Rubenstein, 1993; Rubenstein et al., 1994). Each neuromere is composed of an alar (dorsal) and a basal (ventral) plate. During development, the prosencephalon undergoes further specification splitting into two transverse pro-neuromeric regions, (i) the diencephalon and (ii) the secondary prosencephalon. The diencephalic alar region will be subsequently subdivided into smaller transverse domains known as prosomeres: the pretectum (P1), the thalamus (P2), and the thalamic reticular nucleus (TRN, P3, or pre-thalamus) (Figure 1B), a fundamental structure in the regulation of CT-TC network (Lewis et al., 2015; Crabtree, 2018). The secondary prosencephalon will give rise to the telencephalon and the hypothalamus, including the eye vesicles. From the telencephalon, the cerebro-cortical population will derive to dynamically build the complex texture of CT connections (Puelles et al., 2013; Figure 1C).

CT projection fibers spring up from all areas of the cerebral cortex and every dorsal thalamic nucleus receives its ramifications. The cells implicated in this circuit are pyramidal neurons with spiny dendrites from layer VI of the neocortex (with an ascending apical dendrite that branches in layer IV where it receives monosynaptic feedback from thalamic fibers), as well as from layer V (that branches in layer I with an ascending apical dendrite and extensively connects with collateral axons with the layer III and V) (Alzu’Bi et al., 2019).

The TC connections are subdivided into two classes: core and matrix (Clascá et al., 2012; Harris et al., 2019). The core projections are described as feedforward (driver) and innervate middle cortical layers. The matrix ones, named “feedback” projections (modulator), innervate superficial cortical layers and widely layer I. These projections derive from approximately 60 nuclei (Cassel and de Vasconcelos, 2015) that innervate different cortical areas modulating sensory information.

The above-described CT-TC tangled network is achieved by means of tightly regulated mechanisms such as the existence of “boundaries” and “corridor cells” (Mitrofanis and Guillery, 1993; Bagnard et al., 1998; Grant et al., 2012). Boundaries can be permissive or restrictive and establish precise patterns of guidance molecules (Figure 1C). Indeed, the pallial-subpallial boundary (PSPB—a transient boundary between cerebral cortex and ventral telencephalon domains) and the diencephalic-telencephalic boundary (DTB—lies antero-laterally to the pre-thalamus) operate as turning points where CT/TC axons redirect to invade the thalamus and vice-versa, allowing a correct time maturation of both projections. As postulated by Molnár and Blakemore (1995) in the “handshake hypothesis”, the CT projections are the first to cross the PSPB and reach the internal capsule where they find the rising TC projections. There, proteins like CNR1 (cannabinoid type-1 receptors—also known as CBR1) (Wu et al., 2010) and DRAXIN (Dorsal Repulsive Axon Guidance Protein) (Shinmyo et al., 2015) are essential to guide TC projections toward their cortical targets. Moreover, as observed in the rat model, the subpallium controls the early guidance of TC axons involved in cortical regionalization—regulated by FGF8 and gradients of transcription factors such as PAX6, EMX2, and SP8—and it is sufficient to reorient the TC map within the neocortex (Molnár et al., 1998).

Furthermore, the migratory paths defined by specific cells known as corridor cells (Molnár and Blakemore, 1995; López-Bendito and Molnár, 2003) create the right context to guide the rising network. In particular, the CT projections are aided by cells of the internal capsule, the pre-thalamus, the perireticular nucleus (that coincides with the PSPB and DTB), and by a distinct population of guidepost cells that control the precise pathfinding of TC axons along an internal trajectory within the subpallium, through the release of chemoattractant or chemorepulsive proteins (Bagnard et al., 1998, 2001; López-Bendito et al., 2006). The corridor cells are ISLET1-positive GABAergic neurons that migrate tangentially from the lateral ganglionic eminence into the medial ganglionic eminence and form a cellular “corridor” between the medial ganglionic eminence and the globus pallidus. The interactions between the corridor cells and the TC axons can be modulated through ligand-receptor interactions. For example, in the mouse model, corridor cells express neuroregulin-1 and, in turn, TC axons express the neuregulin-1 receptor ERBB4 on their surface to correctly guide network generation (López-Bendito et al., 2006). Moreover, the cortex exerts a remote control by sending a growth-promoting influence on TC projections when they start to grow out, which becomes growth-permissive when the axons begin to invade the cortex, and later express a “stop signal,” arresting the migration of TC fibers in the layer IV (Métin et al., 1997; Braisted et al., 1999). Lastly, once the network is established, other fundamental regulatory inputs take over.

The TRN plays an important role in modulating the complex communication between the cortex and the thalamus, also by virtue of its anatomical location (Figure 1C). The TRN is innervated by the collateral fibers of layer VI projections and by the collaterals of TC projections (Shine, 2021). The TRN is predominantly composed of an inhibitory GABAergic neural population (more than 80% are parvalbumin-positive cells) that surrounds the main body of thalamic nuclei. It regulates the flow of information between the thalamus and cortex by modulating thalamic activity especially in the antero-medial, ventral-anterior, and medio-dorsal thalamic nuclei, through its inhibitory projections during the transition from wakefulness to sleep, since it generates and modulates sleep spindles (Kaskie et al., 2017). The TRN itself is organized in subnetworks connecting and modulating information from high-order thalamic projection neurons and first-order ones, generating changes in thalamic activity (Crabtree, 2018).

Since the development of the cortex and the thalamus is intricate and the CT-TC circuit establishment needs to be finely synchronized, several insults in diverse crucial spots could lead to incorrect regionalization, impaired neuronal migration, and aberrant innervation of the involved areas. All these alterations may lead to a series of neurodevelopmental disorders, among which SCZ, autism spectrum disorders, and bipolar disorder have increasingly been associated with severe CT-TC network disturbances, which have been coupled with several clinical outcomes (Anticevic et al., 2014; Castelnovo et al., 2015; Murray and Anticevic, 2017; Hwang et al., 2022). Intriguingly, SCZ also appears to be specifically linked to TRN alterations, which could play a pivotal role in overall CT-TC network disruption. Indeed, reduced TRN inhibition is expected to trigger a less filtered thalamic relay and motor information to the cortex during wakefulness and a reduced burst firing, which is necessary for sleep spindle modulation (Ferrarelli and Tononi, 2011; Manoach et al., 2016; D’Agostino et al., 2018; Thankachan et al., 2019).

## Animal models to study cortico-thalamic development

Most of the current knowledge about the complex CT development relies on studies performed on animal models. For instance, fate mapping studies and more advanced techniques of RNA sequencing from specific areas provided information about the cellular and molecular identity of developing thalamic neural populations (Kim et al., 2020, 2022; Puelles et al., 2021). Indeed, Puelles et al. (2021), by exploiting transgenic reporter mouse lines, demonstrated unique features of TRN architecture, a fundamental structure in regulating the CT-TC circuit activity. Indeed, they mapped for the first time the prethalamic neural population and its cellular complexity (Puelles et al., 2021). They surprisingly noted distinct immunoreactivity for the *DLX* gene family, essential for the specification of GABAergic cells (Anderson et al., 1997), in the prethalamic area. Thanks to this approach, Puelles and colleagues proposed a new resulting model that subdivides the studied region into four subareas (rostro-caudally and dorso-ventrally), as well as a tripartite radial stratification, with a specific molecular profile. Thus, the study denotes a more complex scenario for prethalamic patterning as well as new insights into the CT-TC connections, since the first one must be guided through this heterogeneous area. Another important contribution is represented by the study of Kim and colleagues who performed single-cell transcriptomic analyses that revealed evolutionary conservation among major neural precursors with distinct hypothalamic and thalamic specification (Kim et al., 2022). In this study, the authors used hypothalamus from the large and accessible chick embryo at six different developmental timepoints, ranging from hypothalamic induction and regionalization, to early neurogenesis (Kim et al., 2022). By profiling gene expression through single-cell RNA-sequencing, the authors identified both known and novel markers that, when validated with multiplexed hybridization chain reaction analysis, allowed them to define and link major spatial domains at each stage (Kim et al., 2022). They clarified the organization of the nascent hypothalamus highlighting conserved key gene expression patterns between neuronal precursor clusters in chicken, mouse, and human developing prethalamus and hypothalamus (Kim et al., 2022).

All these cardinal findings confirm the importance of animal studies, which still represent a valid model to disclose the molecular and physiological mechanisms regulating the complex CT-TC network in a living organism.

Moreover, the CT-TC system has been widely used to study axon guidance mechanisms, decode the components implicated in the development of cortical circuits, comprehend the development of sensory systems (Blakemore and Molnar, 1990; López-Bendito, 2018), and investigate the mechanisms involved in anatomical and functional circuit plasticity following a sensory loss (Kwan and Dan, 2012; Chambers et al., 2022).

The latest studies employed cutting-edge tools to selectively manipulate specific cell types belonging to specific neural populations and sub-populations combined with electrophysiology. These approaches have disclosed the opportunity to directly examine the role of CT pathways in behavior regulation (Alcaraz et al., 2018; Antunes and Malmierca, 2021; Fratzl and Hofer, 2022; Spacek et al., 2022). For instance, Alcaraz et al. (2018) evaluated the functional implication of selected CT and TC pathways connecting the dorsomedial prefrontal cortex (dmPFC) and the mediodorsal thalamus (MD) in rat behavior. By applying a chemogenetic approach to inhibit projection-defined dmPFC and MD neurons during a specific learning task, they illustrated that CT and TC pathways differentially support goal attributes. The study indicates that the antiparallel flow of information within TC circuits may convey qualitatively distinct aspects of adaptive decision-making and highlight the importance of the direction of information flow within neural circuits.

Similar approaches have been also applied to study neurodevelopmental disorders associated with CT-TC network dysfunction (Mancini et al., 2020; Jiang et al., 2021; Breuer and Krieger, 2022). Abnormal connectivity between the thalamus and cortical areas is an acknowledged feature of SCZ (Chen et al., 2019), as well as the volume reduction of prefrontal cortical regions targeted by TC projections (Lewis, 2012; Zhang et al., 2021). Moreover, several studies also identified alterations in sleep spindles—generated and modulated by the TRN in the CT-TC network—as promising inheritable endophenotype of SCZ (Ferrarelli et al., 2007, 2012; D’Agostino et al., 2018).

Animal models paved the way to dissect and unveil human neural ontogenesis, giving us the possibility to explore human brain complexity. Despite the large application and advantages, some limitations are present in terms of accuracy in mirroring human physiological and pathological phenotypes. For this reason, new technologies are emerging with the purpose to simulate, as far as possible, the human system.

## Shifting borders—Exploring cortico-thalamic development through *in vitro* and *in silico* models

Human cell-based *in vitro* models are considered a new frontier to disentangle cellular and molecular events of CT development, and its deviations, in humans.

For instance, traditional *ex vivo* methods such as organotypic cultures have been exploited as a valid system to investigate human neurodevelopmental alterations of specific networks. Indeed, organotypic brain slice cultures represent a physiological three-dimensional model of the brain that can be interrogated to define the impact of genetic and environmental insults and pathophysiological responses (Onorati et al., 2016; Grønning Hansen et al., 2020; McLeod et al., 2022). This method, indeed, allows the culture of neural complex tissue preserving the architecture and cell interactions, even if the obtainment and long-term maintenance of the culture may be challenging.

Nowadays, 2D and 3D neural cultures derived from human pluripotent stem cells (hiPSCs) (Dell’Amico et al., 2021) provide a key tool to comprehend the complex CT networks. Moreover, the application of targeted neural differentiation protocols with a specific combination of morphogens allows the generation of very specific neural populations from different brain areas (Kirkeby et al., 2012; Xiang et al., 2019, 2020). For instance, Kirkeby et al. (2012) generated different region-defined neural progenitors through the addition of CHIR99021, an agonist of canonical WNT signaling that induces efficient dose-dependent specification of the telencephalic and diencephalic fates. Intriguingly, it is also possible to generate 3D organoid structures of specific brain areas to obtain a more reliable system that recapitulates the cellular complexity as well as the 3D architectures of the brain region of interest *in vitro*, including the thalamus (Xiang et al., 2019, 2020).

While 2D neural cultures can be useful for the identification of molecular pathways and the study of neural population composition (Kanagasabapathi et al., 2012), brain organoids represent a meaningful tool to recapitulate and investigate neural ontogenesis but also to dissect network establishment. For instance, Xiang et al. (2019), generated and characterized cortical and thalamic organoids. They assembled these organoids into an innovative 3D co-culture system, defined as assembloid (Xiang et al., 2019). This approach offered a platform to understand and mirror circuit organization and related disorders. Indeed, organoids better re-create the 3D interactions occurring in the developing human brain.

An important goal in this field was accomplished by Revah et al. (2022). They transplanted hiPSC-derived cortical organoids in the primary somatosensory (S1) area of the developing cerebral cortex of early-postnatal rats. They examined human neural differentiation and function *in vivo*, demonstrating that human cortical neurons mature, receive host thalamic connections, and engage host circuits to control host behavior (Revah et al., 2022). This approach proposes not just a merely developmental study platform but also a useful tool for detecting circuit-level phenotypes in patient-derived cells that can be perceived only by means of this model. Despite some limitations of organoids, including low reproducibility, incomplete layering, and anatomical parcellation, they provide an opportunity to model unique features of the human brain (Di Lullo and Kriegstein, 2017). Thanks to the iPSC technology, the coexistence of the *in vivo* and *in vitro* models to explore human neural circuitry becomes more accessible everyday.

*In silico* techniques can be integrated to deeply understand the composite brain circuitry, including computational models such as the NEURON simulator and the NetPyNE tool (Dura-Bernal et al., 2019). These approaches match *in vivo* and *in vitro* experimental findings, allow the simulation of realistic inputs/outputs considering the original cell morphologies and electrophysiological responses, and generate predictions of the dynamics and functions of microcircuits (Griffiths et al., 2020; Borges et al., 2022). These avant-garde techniques can be also exploited to further explore the system from the inside, allowing the analysis of neural properties thus controlling cell-specific connections and its implication inside a network. For example, Huang et al. (2022) applied this innovative approach to reconstruct the barrel cortex to *in silico* replicate properties of touch representations. This model allows to unveil new principles of information processing through the identification of circuit components and the influence of connectivity on network behavior.

**FIGURE 2:**
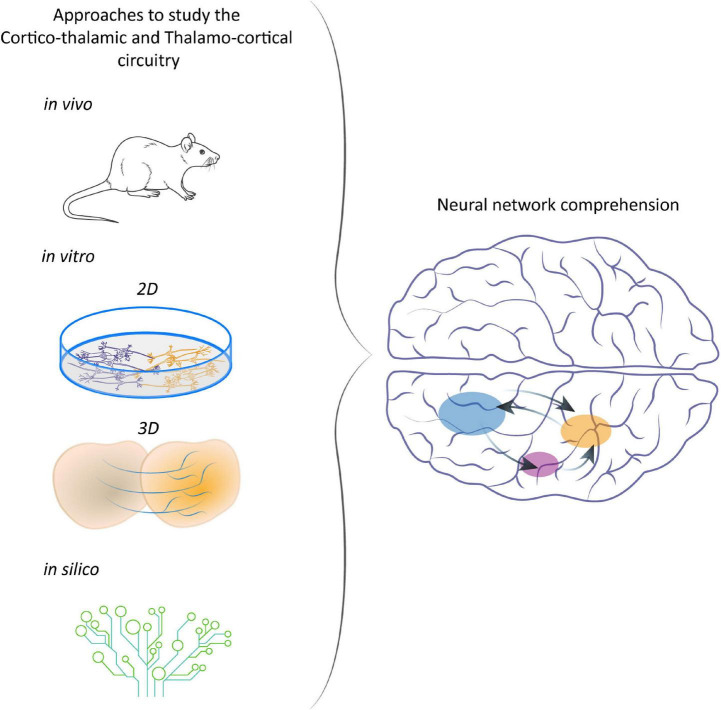
Different approaches to study CT-TC network. From animal models to *in vitro* and *in silico* systems to understand the development of CT networks and the consequences of their disruption.

The synergic application of all these different techniques made it possible to add another step toward the understanding of CT-TC neural circuitry (Figure 2) and thus of its possible deviations.

## Conclusion

Access to the developing human brain to study the mechanisms of CT-area specification and the establishment of its complex neuronal connections remains a great challenge. Aberrant neurodevelopmental and psychiatric disorders appear to be strictly interconnected and should be investigated from different points of view. In SCZ, autism spectrum disorders, and bipolar disorder, physiological neural networks are altered leading to dysregulated brain circuit establishment and function. In particular, the CT-TC circuitry, which modulates essential tasks such as sleep, learning and memory, and even consciousness, may be severely affected in SCZ. Unveiling the ontogenesis and organization of specific CT-TC neural networks through cutting-edge platforms may significantly advance our understanding of several neurodevelopmental and psychiatric disorders. In this field, classical developmental studies performed in animal models provide the knowledge to instruct and generate *in vitro* organoids and assembloids to better mimic human-specific network formation/maturation. Moreover, the combination of these platforms with the iPSC technology offers new approaches to uncover brain development and function and clarify neuron-neuron interaction in complex networks.

## Author contributions

MMAS and MO drafted the manuscript. MMAS designed the figures. MO, CD’A, AD’A, and LC revised the manuscript, provided the guidance, and conceptual support. All authors contributed to the article and approved the submitted version.
